# 2D Memory Transistors for Neuromorphic Computing

**DOI:** 10.3390/ma19214710

**Published:** 2026-11-05

**Authors:** Judy Wu, Andrew Shultz, Saad Alzahrani, Ryan Goul

**Affiliations:** Department of Physics and Astronomy, University of Kansas, Lawrence, KS 66045, USA; andjshultz@ku.edu (A.S.); alzahrsa@ku.edu (S.A.)

**Keywords:** memory devices, 2D electronics, neuromorphic, artificial intelligence

## Abstract

The boom in artificial intelligence has driven in-memory computing for high speed and power efficiency. Motivated by the need, neuromorphic computing (NC)—mimicking biological spike neural networks—ies a promising alternative that requires artificial neurons and synapses to construct NC circuits. 2D memory transistors have received intensive interest in recent research for NC applications as both artificial neurons and synapses with additional advantages for high device density and energy efficiency. This review intends to highlight recent progress made in the exploration of 2D memory transistors and their implementation into electrical and optoelectronic NC circuits. Discussions of remaining challenges and future perspectives are also provided.

## 1. Introduction

### 1.1. Overview of Neuromorphic Computing

The AI boom [1,2] has brought in a new age of technological advancement, but with its arrival came disruption to the computing industry in the form of shortages of the energy-efficient computing hardware needed in data centers and the enormous power grid strains caused by AI datacenters. To tackle the problem of machine learning for AI, graphical processing units (GPUs) were repurposed to take advantage of their high degree of parallel computing in addition to the creation of new purpose-built hardware. These new pieces of hardware implemented alternative computing schemes such as neuromorphic computing, which attempts to emulate biological neurons and synapses [3]. The human brain contains 10^11^ neurons that communicate via 10^15^ synapses and operates on a mere 20 watts of power [4]. To take advantage of the parallel nature of the human brain and the in-memory computation that allows the brain’s extremely efficient operation, neuromorphic computing researchers have created their own sets of artificial synapses and neurons to enable highly parallel inference operations for machine learning [5]. Large-scale neuromorphic computing (NC) chips with millions of neurons and hundreds of millions of synapses, such as BrainScaleS [6], TrueNorth [7,8], NorthPole [9,10], Loihi [11], have been successfully built due to the maturity of complementary metal oxide semiconductor (CMOS) technology. However, the energy efficiency of these NC chips is of the order of 10^10^ to 10^12^ synaptic operations per second per watt (SOPS/W) [12], which is several magnitudes lower than that of human brain (10^12^ to 10^14^ SOPS/W), where hundreds of billions of neurons and thousands of trillions of synapses operate [13]. Other NC chips with architecture based on an array of six-transistor static random access memory or 6T-SRAM bit cells show high energy efficiency, up to ~10^15^ SOPS/W [14]. However, the SNR of this type of chip is inversely proportional to the number of bit cells. To ensure reasonable SNR, the scale, that is the number of bit cells, is limited to several megabytes [15]. Therefore, it is imperative to find new energy-efficient and scalable electronic devices that can mimic biological neural systems.

Neuromorphic computing contains a multitude of different architectures, but at its most general there is the artificial neural network (ANN), which uses multiple layers of weights represented by tunable memory units, accompanied by neuronal activation functions between each neural network layer to provide abstraction and allow the extraction of features present in datasets leading to the classification of data into different classes [16]. During network training to recognize specific patterns or items, the network receives pre-categorized data from a training set while the output of the ANN is monitored. Based on the errors in classification, this information is used to propagate backwards through the neural network and adjust weights to more accurately classify inputs. However, in order to test each input piece of data in a physically represented ANN such as GPUs or custom-built circuits, all weights must be biased and undergo vector matrix multiplication (VMM), which consumes significant amounts of energy as the complexity and size of the data increases. In ANNs, these multiplying and accumulating steps during inference are considered the aforementioned synaptic operations. As an alternative to this, for sparser datasets such as event-driven camera data, where many weights are irrelevant for a given dataset, the spiking neural network (SNN) has been investigated [17]. SNNs operate using a similar weight scheme as ANNs, but the inputs, interlayer communications, and outputs are communicated using short voltage spikes. As the network receives spikes from the relevant inputs, their amplitudes are attenuated by network weights before reaching a spiking neuron, which is an SNN version a of a synaptic operation. These neurons are typically designated to have leaky, integrate, and fire (LIF) characteristics, since they receive the pulses, build a neuron-like potential, and fire when a threshold is reached. This results in a sparse activation of weights and neurons in SNNs as opposed to dense VMM in ANNs, which can drastically reduce power consumption, especially in the case of event-driven data such as event cameras or sound recordings.

**Figure 1:**
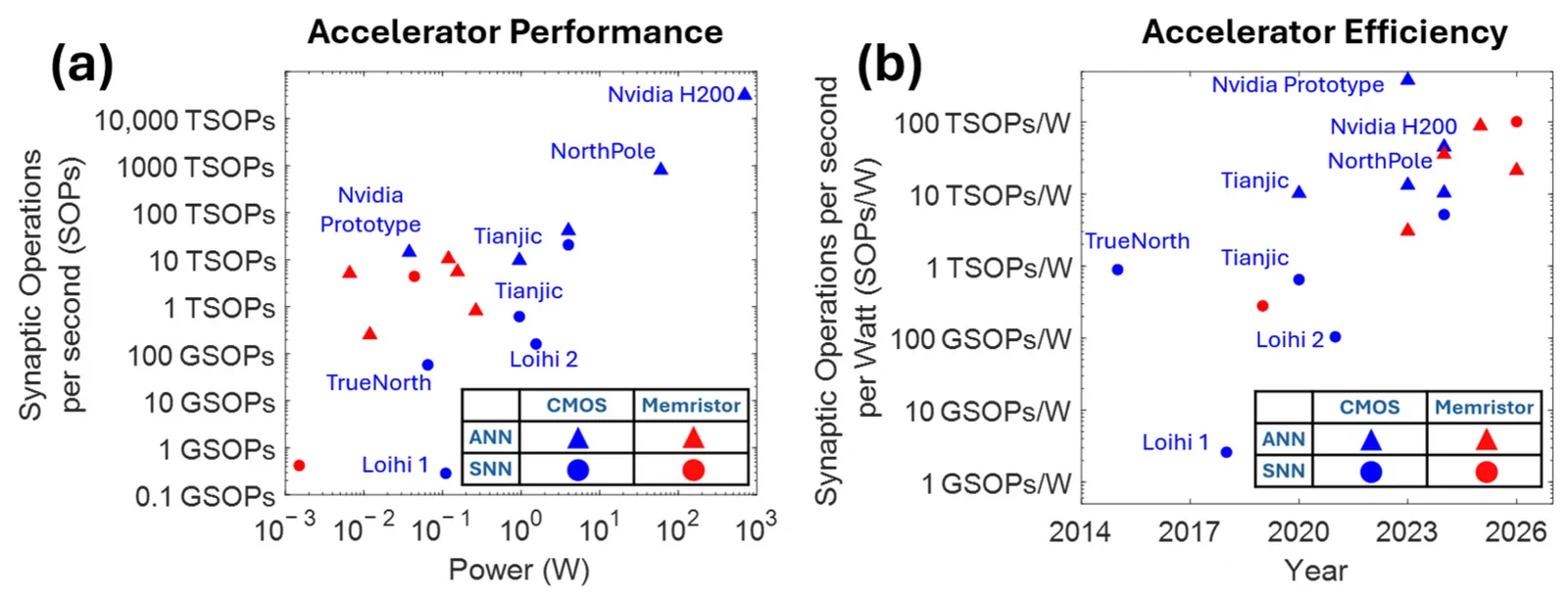
SOPs Comparison: (**a**) Comparison of total system power versus system performance (giga or tera synaptic operations per second) for CMOS ANNs [9,18,19,20,21], CMOS SNNs [8,11,18,19,22], Memristor ANNs [23,24,25,26,27], and Memristor SNNs [28,29]. (**b**) Evolution of accelerator energy efficiency (synaptic operations per second per Watt) through time. CMOS SNN and ANNs are normalized to 1-bit energy usage and performance according to the highest reported performance. Efficiency is assumed to be using optimal settings for the given architecture and dataset.

Researchers and the semiconductor industry at large have probed different algorithms, architectures, and novel components to achieve high performance in machine learning tasks. However, this often comes at a steep power cost (Figure 1a) [8,9,11,18,19,20,21,22,23,24,25,26,27,28,29]. For example, Nvidia’s H200 GPU and IBM’s NorthPole accelerator achieved performance in the hundreds to thousands of TSOPs range, but must drive their systems with proportionally higher powers [9,10,21]. Although the SNN architecture may provide some relief to power usage through sparse weight activation, this only applies to certain subsets of problems that are asynchronous event-driven datasets, whereas performance is equal or lower compared to ANNs when processing regular datasets.

Recent research has turned toward optimizing energy-efficiency by utilizing in-memory computing with nonvolatile (zero steady power input to maintain memory) synaptic weights and switching from digital to analog neurons to reduce power usage (Figure 1b) [28]. Energy usage and performance in CMOS tends to scale linearly with the bit accuracy due to the added transistor count to represent multiple levels of a weight, costing speed and energy to add accuracy to a machine learning task. Memristive systems, however, represent weights with resistive memory computational cells, in which single analog devices represent multi-bit states. For that reason, direct correlation with bit-level accuracy and per-synapse energy usage must contend with the DAC/ADC energy overhead and how efficiently an array of artificial synapses is utilized relative to number DAC outputs and ADC inputs [30]. For this reason and the fact that many different datasets may result in suboptimal utilization of weight arrays in these accelerators, the reported values in Figure 1 are normalized to 1-bit where applicable, and it is assumed the authors reported their highest performance, most efficient tests as their key results, regardless of the problem set.

To create these neural network weights, nonvolatile memristors were implemented in both ANNs and SNNs. Memristors are devices that can take on multiple conductance states depending on input voltage or other stimuli, and can represent multiple memory levels as artificial synapses [31].

Artificial LIF spiking neurons were created to emulate the efficient pulse integration and threshold firing mechanisms observed in biological neurons. Both artificial neurons and synapses were fabricated using the mature CMOS transistor paradigm. However, to enable these devices to emulate rich biological dynamics, six transistors are typically required for synaptic functions and up to eighteen for neuronal functions, thereby limiting chip density [32]. Therefore, in order to solve a looming density problem in machine learning accelerator chips, it is extremely important to explore and develop new types of artificial neurons and synapses capable of implementing these rich operational dynamics in a single or few integrated devices. To this end, 2D atomic materials provide promising alternatives to matured CMOS technology. It should be mentioned that many excellent reviews have been published to cover the exciting progress made in the last two decades on the synthesis, characterization, and applications of 2D atomic materials and their van der Waals heterostructures [33,34,35,36]. However, 2D memory transistors represent an emerging topic in the research of 2D materials, targeted specifically at realizing characteristics of artificial neurons and synapses for neuromorphic circuits. Considering it has not been covered with adequate depth and breadth in literature, this review aims to highlight the recent progress achieved in this important research area together with a discussion on future perspectives.

### 1.2. Brief Overview of 2d Materials and Van Der Waals Heterostructures

The isolation of monolayers of graphene [37,38] in 2004 sparked intensive research into 2D materials [39] due to their unique electronic structures, which are enabled by 2D quantum confinement, inherent flexibility, transparency, and a high surface-to-volume ratio for application in next generation electronics and optoelectronics [40,41,42,43,44]. Common 2D materials include: graphene, transition metal dichalcogenides, hexagonal boron nitride, black phosphorus, MXenes, etc. [45,46]. Further benefits can be realized through the stacking of different 2D materials to form van der Waals heterostructures (vdWH), named for the weak van der Waals force between layers (in contrast to the strong in-plane atomic bonding) to achieve a favorable electronic structure for charge transfer. For example, the linear dispersion in a so-called Dirac cone electronic structure for graphene results in massless fermions (both holes and electrons) with an extraordinary Fermi velocity; the theoretical limit is 10^6^ m/s and hence the carrier mobility could be up to 10^6^ cm^2^/V-s [38]. The high surface-to-volume ratio of 2D materials means that nearly the entire volume of the material is affected by the interfacial interaction, in contrast to bulk materials where this effect is often negligible. Stacking 2D materials into vdWH eliminates the lattice matching and chemical composition compatibility requirements of traditional thin film epitaxy, allowing for the exploration of novel electronic structures [47,48,49]. Some properties realized from these unique structures include ferroelectricity, ferromagnetism, superconductivity, etc. [50,51,52,53,54,55]. Additionally, the stacking angle of different layers can produce unique interface moiré patterns, resulting in the new field of twistronics [56,57]. A chief concern for applications of vdWHs is interfacial purity, where trace amounts of contaminants can serve as charge traps or an insulating layer, rendering a device faulty or inoperable by preventing desired vdW interfaces from forming to enable the desired interface-coupling. A number of surface cleansing techniques have been employed, including plasma cleaning, thermal and vacuum annealing, and nondestructive light irradiation, with applicability dependent on the specific material tolerances [58,59,60]. The benefits of vdWHs have seen applications in field effect transistors, photodetectors, photovoltaic cells, light-emitting diodes, spintronics, and neuromorphic devices [40,41,44,61].

### 1.3. Recent Progress in 2d Field Effect Transistors

**Figure 2:**
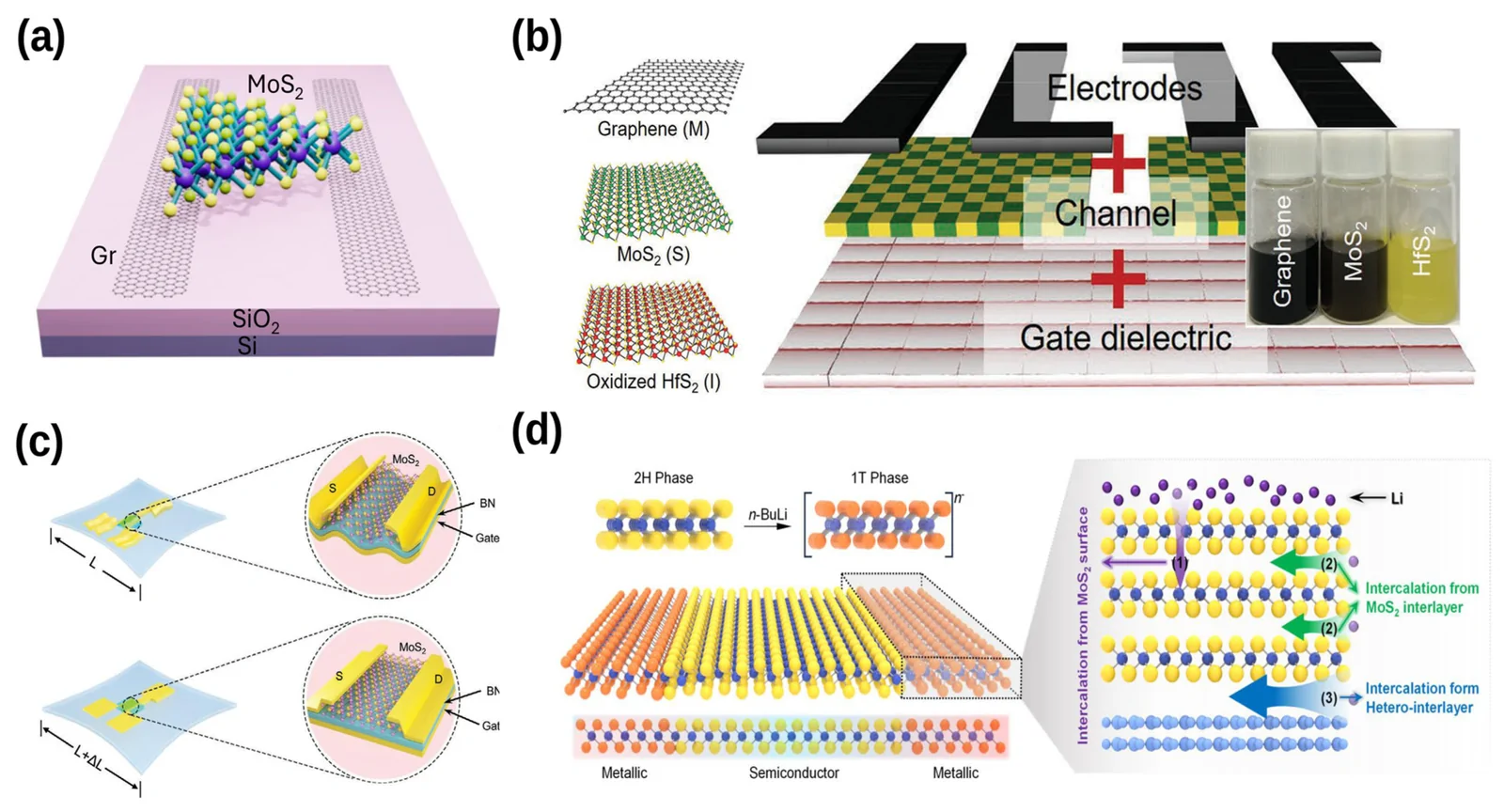
2D FETs: (**a**) All CVD synthesized 2D vdWH FETs. (**b**) All-solution processed 2D vdWH FET [66]. (**c**) Flexible 2D vdWH FET [66,67]. (**d**) 2D vdWH FET with tailored intercalation pathways [68]. Copyright 2024, Springer Nature. Adapted under CC BY 4.0 license [65]. Copyright 2021, John Wiley and Sons. Adapted with permission [66]. Copyright 2022, John Wiley and Sons. Adapted with permission [67]. Copyright 2024, John Wiley and Sons. Adapted under CC BY 4.0 license [68].

Two-dimensional field-effect transistors (2D FETs) have emerged as promising candidates for continued transistor scaling because their atomically thin channels provide strong electrostatic gate control and suppress short-channel effects. Graphene FETs exhibit exceptionally high carrier mobility, approaching the theoretical limit of 200,000 cm^2^ V^−1^ s^−1^ in high-quality suspended devices [62]. Semiconducting 2D FETs provide an additional advantage by combining atomic-scale channel thickness with finite band gaps resulting in high I_on_/I_off_ ratios. Monolayer MoS_2_ FETs, for example, exhibit a direct band gap of approximately 1.8 eV, reported field-effect mobilities approaching 700 cm^2^ V^−1^ s^−1^, and I_on_/I_off_ ratios of 10^7^–10^8^ [63]. These characteristics become increasingly important as silicon FETs approach their physical scaling limits. Therefore, 2D FETs preserve a highly crystalline channel at atomic thickness. Wu et al., for example, demonstrated bilayer WSe_2_ FETs with channel lengths of less than 100 nm and performance comparable to silicon transistors at similar operation voltages [64]. Advances in fabrication processes have demonstrated scalability of 2D vdWH FETs, with recent works in chemical vapor deposition or CVD grown (see Figure 2a) [65] and all-solution processed 2D vdWH FETs (see Figure 2b) [66]. Recent works in flexible/wearable electronics have also shown promising progress, with Li et al. reporting a stretchable 2D FET based on wrinkled MoS_2_ (see Figure 2c) [66,67]. Additionally, Yu et al. demonstrated that high-quality edge contacts for lateral 2D vdWH FETs can be established with tailored lithium intercalation pathways (see Figure 2d) [68].

2D FETs are increasingly being developed as memory transistors (memFETs) for neuromorphic and in-memory computing. Conventional all-CMOS implementations may require multiple transistors to construct a single artificial neuron and synapse, increasing circuit volume, power consumption, and interconnection complexity [32]. In contrast, a single 2D memFET can combine transistor operation, dynamic memory, and analog conductance modulation, potentially reproducing neuronal or synaptic functions, exhibiting excellent potential for neuromorphic computing.

## 2. 2D MemFETs for Neuromorphic Computing

### 2.1. Neuromorphic Computing Requirement on 2D MemFETs

#### 2.1.1. Artificial Synapse Requirements

**Figure 3:**
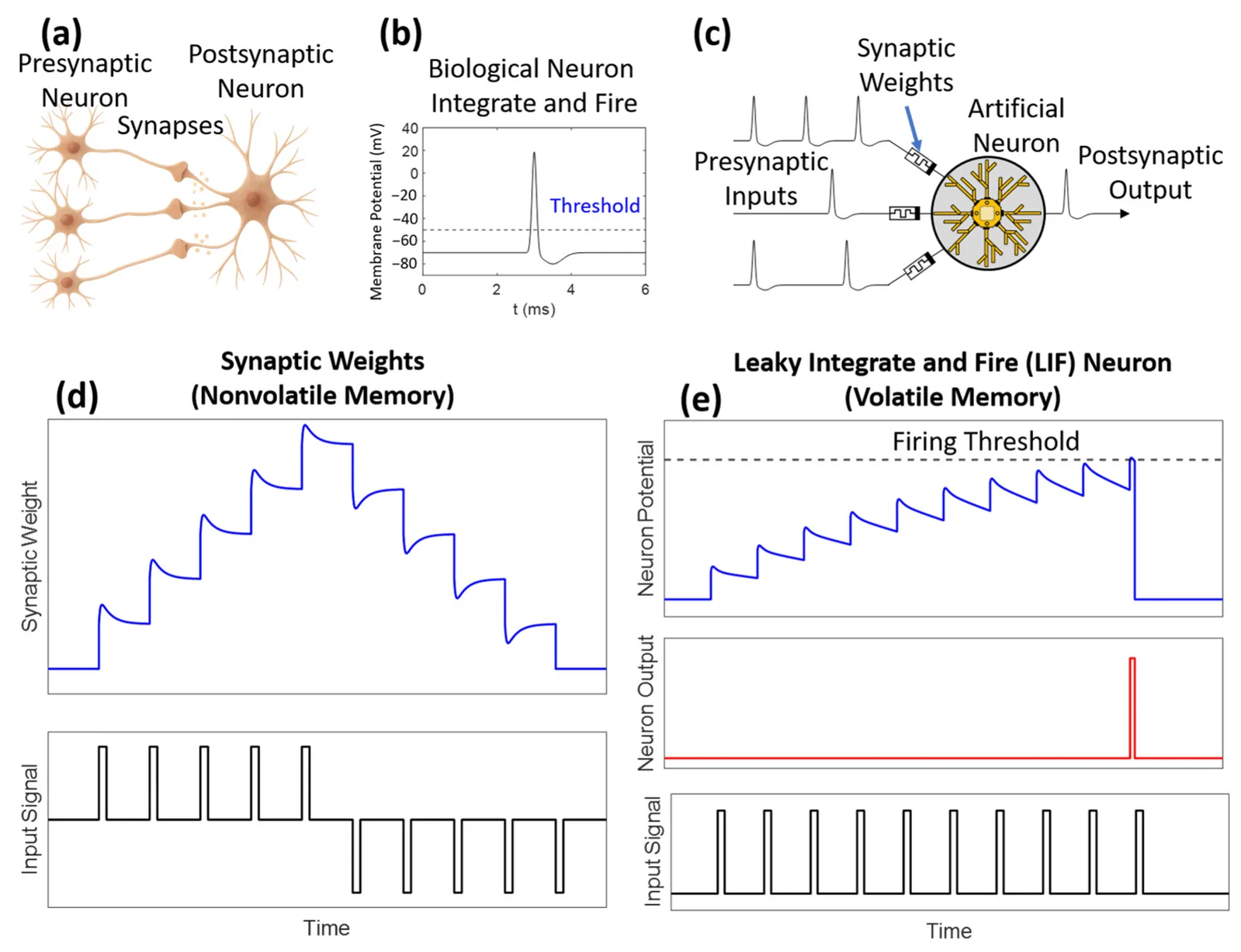
Neuron/Synapse Diagram: (**a**) Biological presynaptic neurons, synapses, and postsynaptic neurons. (**b**) Biological neuron integrate and fire approximation. (**c**) Neuromorphic SNN diagram with volatile artificial neuron receiving attenuated input signals through resistive synaptic weights. (**d**) Example of a nonvolatile artificial synapse whose weight is increased by positive pulses and decreased by negative. (**e**) Example of a leaky integrate and fire artificial neuron which integrates input pulses and fires an output signal after reaching a threshold.

Biological neuron synapse dynamics are the same decentralized parallel architecture upon which artificial neurons and synapses are modelled (Figure 3a). Through depolarization and relief of Mg^2+^ blockage of receptors, new Ca^2+^ may flow in thereby incrementally strengthening synaptic weights. Signals from presynaptic input neurons sent through synapses may be integrated by postsynaptic neurons through changes in neuron potential to create an efficient signal propagator that only fires if and when sufficient potential has been accumulated within a short time (Figure 3b) [69]. One of the key pain-points that 2D memFET as an artificial synapse may address is the amount of memory states representable in a single device. In machine learning accelerator hardware, the resolution of weights can negatively affect network accuracy. These weights are commonly tuned through light or positive/negative gate voltage pulses (Figure 3d), but it is necessary that the change in the channel conductance be mostly linear so that training rate and neural network accuracy do not suffer due to over/undershooting. In 2D memFETs, this change in the channel conductance is typically a result of trapped weights. In the case of 2D memFET as an artificial synapse (Figure 3c), these changes are typically governed by channel conductance charge in a floating gate [70,71], ionic layers building on an FET channel [72], or polarization of a ferroelectric dielectric gate layer [73]. The range of the tunable channel conductance should be significant enough that the resulting conductance states can clearly be distinguished, but as long as there is a nonlinear *IV* response, the problem of sneak path currents present in normal memristor arrays can be avoided [74]. Devices used for synaptic weights are typically nonvolatile, as the same weight level must be maintained, sometimes for hours, while performing ANN/SNN inference operations, and do not require outside circuitry to perform as analog memory. Preferably, these artificial synapses would be resistant to heat, with only minor changes in channel conductance with heats up to 100 °C, which is not uncommon in many computing environments. Comparatively, other types of memristors—such as phase change memory, which alters the phase of a switching material, and redox memristors, which force ion migration back and forth through a switching layer—have shorter lifespans, in the range of 10^6^ cycles in most devices. Partially analog switches increase this lifetime considerably [5]. FeFETs and memeFETs used as synapses typically have a lifespan of 10^3^ to 10^8^ cycles.

#### 2.1.2. Lif Neuron Requirements

Artificial LIF neurons are critical to SNN signal propagation and event classification due to their event driven operation via input pulse integration. As signals arrive at the artificial neuron, they are integrated, eventually reaching a threshold and causing a pulse to be fired either due to the sudden decrease in conductance resulting in a voltage spike output (Figure 3c,e), or through an exterior means to generate a pulse once a threshold current level is reached in the 2D memFET [75,76]. The leaky characteristic describes how this neuron potential decays over time if no new inputs are received, which in event-based data means that unless events occur in a short time frame, their input spikes will not be enough to trigger the threshold and send a spike further into the network. Control over the firing threshold and leakage rate of an LIF neuron is extremely important to avoid incorrect neuron firing during intermediate or classification steps. External circuits automate resetting spike generation using traditional components such as comparators and parallel/series capacitors to reduce/increase the leak-rate of the neuron potential. Digital circuits enable a refractory period immediately after neuron firing to prevent refiring. Volatility and spike generation are the most difficult to intrinsically engineer into devices with only a few diffusive and mott transition memristors capable of this but still requiring a parallel capacitor to control leakage rates [75,77,78].

#### 2.1.3. Uniformity and Reproducibility

In order to create a full network of artificial synapses, many high-resolution weights are required, and they must respond in a similar and reproducible manner. During the weight tuning phase of training the neural network, identical voltage pulses are sent to each device to control its conductance level, since in scaled up networks having different weight update pulses device to device would require a large amount of overhead circuitry, costing chip real estate, energy, and time, meaning that the array of artificial synapses must have reasonably low variability to maintain higher accuracy. Artificial neurons must not only fire consistently given the same inputs, but their potential building, leaky characteristic, and firing output must be similar across the array to ensure that specific features of the data do not get muffled and that specific classes are not favored over others, thus biasing the classification outputs. This non-uniformity in neuron characteristics can be dealt with by selecting an overlapping channel conductance range to operate multiple neurons in, even if their on/off ratios and minimum/maximum currents do not perfectly match.

### 2.2. Physics of Memory Effect in 2D FETs

Memory devices have been widely adopted in modern microelectronics as primary or internal memory to provide fast and temporary memory during computing and secondary or external memory for long-term data storage. There are many mechanisms used to generate memory states in these devices, including different resistance states, magnetic ordering, etc. For example, thin film memristors can exhibit low resistance and high resistance states (LRS and HRS) via the generation and rupture of a conducting filament repeatedly upon the application of electric voltages of opposite polarities, offer high energy efficiency (>10^15^ SOPS/W) [79,80], fast switching speed (>10 GHz) [79], and excellent size scalability (down to a lateral dimension of 2 nm and thickness in sub-2 nm) [79,81,82,83,84,85,86]. Different resistance states can also be generated in ferroelectric and magnetic tunnel junctions by controlling the quantum coherence tunneling between the electric or magnetic dipoles in the two electrodes at different dipole orientations [87,88,89]. Implementing memory states in 2D devices is an emerging topic, with exciting progress made recently in charge-based and resistive switch mechanisms [90,91]. Gates have been commonly employed to induce and control memory states, and ionic transport may provide an interesting alternative with implemented controls. Monolayer 2D atomic sheets have the highest surface to volume ratio, which may allow interfaces in vdWH structures to provide unique engineering approaches to manipulating 2D monolayer FET channel conductance. Among various interface tuning reported, interface charge trapping on defects is a primary approach [91]. While memory states can be generated, controlling the memory states with the required reproducibility could be hindered by uncontrollable charge trapping mechanisms associated to interface defects. In order to address this issue, the implementation of ionic liquid (or solid) gates was recently explored by a few groups. This requires the formation of a molecular electric double layer (EDL~1.0 nm in thickness) at the interface with the 2D memFET channel. Memory states can be generated by controlling the electric dipole concentration in the EDL, which provides superior reproducibility of memory states because EDL formation relies on a weak interface interaction between the ionic gate and 2D materials. This means such an EDL may be applied broadly to a variety of 2D atomic layers and the interface interaction may be designed by selecting a suitable ionic gate and the 2D channel material to form a desired EDL. Besides memory state reproducibility, an additional advantage of this approach is in its scalability to large arrays of memFET with high device yield and uniformity.

## 3. Recent Advances in 2d Memory Devices and Circuits

### 3.1. Research and Development of 2d Memfets

#### 3.1.1. All Electrically Operated 2d Memfets

**Figure 4:**
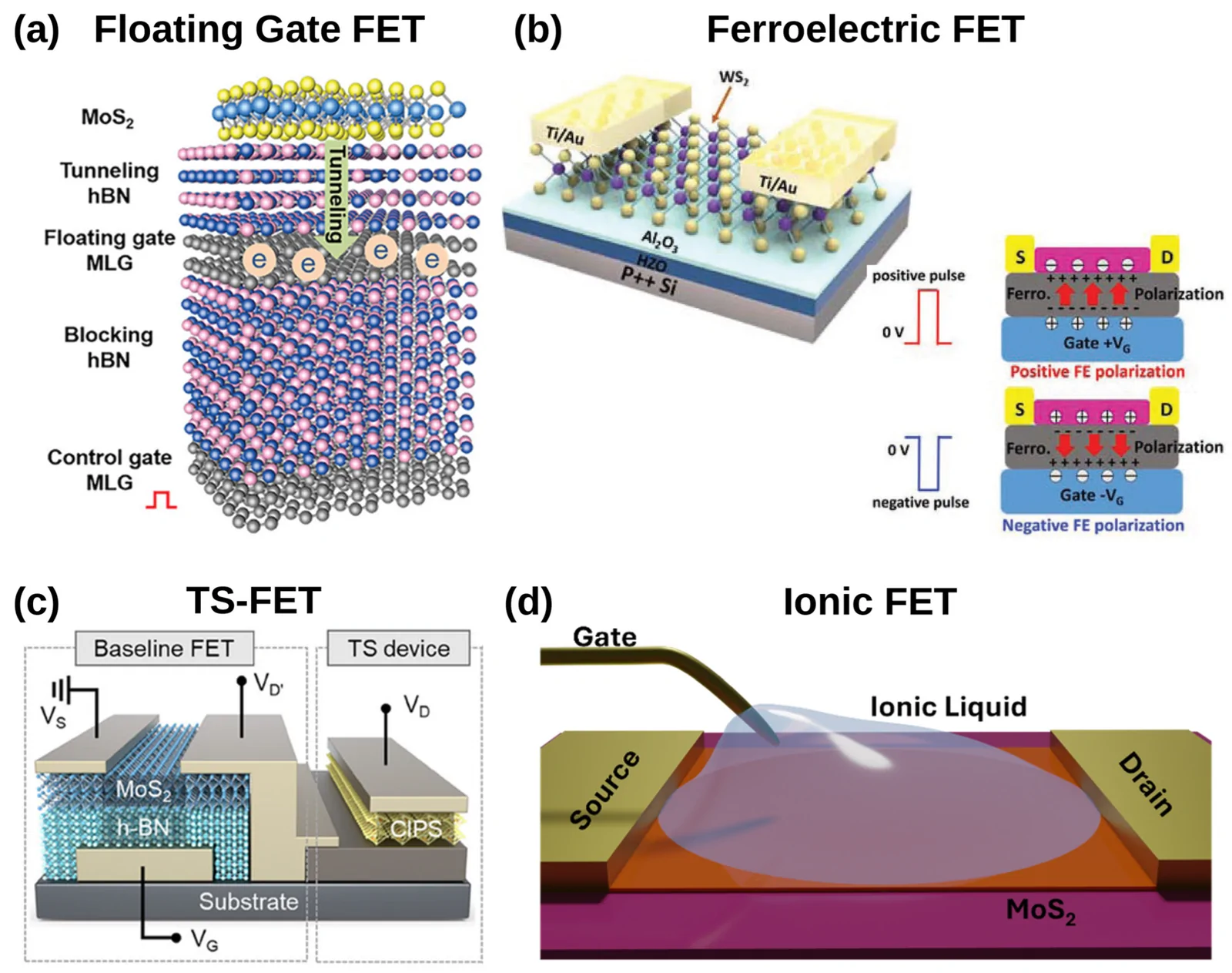
Electrically Operated 2D memFETs: (**a**) Structure of memory floating gate FETs. (**b**) Structure and mechanism of memory ferroelectric FETs. (**c**) Structure of memory TS-FETs. (**d**) Structure and mechanism of memory ionic FETs. Copyright 2024, John Wiley and Sons. Reproduced with permission [103]. Copyright 2020, John Wiley and Sons. Reproduced with permission [95]. Copyright 2025, John Wiley and Sons. Adapted under CC BY-NC 4.0 license [102].

In all electrically operated 2D memFETs, both SET and RESET are induced by electrical pulses. In many devices, voltage pulses are applied to a control gate, and the required polarity, amplitude, and duration depend on the material stack and underlying memory mechanism. One well-established implementation is the floating-gate transistor. First introduced in silicon-based CMOS structures, the floating-gate architecture uses a conductive charge-storage layer isolated between a tunneling dielectric and a blocking dielectric. The tunneling layer permits charge injection during SET operation, whereas the blocking layer suppresses charge loss and supports nonvolatile retention (Figure 4a). 2D atomic materials extend this architecture to van der Waals stacks with thin channels and clean interfaces, offering a possible route to improved electrostatic scaling [92,93]. Li et al. reported a floating-gate device comprising a graphene–black phosphorus heterojunction, a 14.5 nm h-BN tunneling barrier, and a separate graphene floating gate on 300 nm SiO_2_/Si, with Si serving as the control gate. Control-gate pulses of −20 and +20 V, each with a duration of 300 ms, produced the SET and RESET, respectively [94]. Retention was measured for 1200 s, and extrapolation of the charge-decay behavior predicted that approximately 50% of the trapped charge would remain after ten years. Despite this strong projected retention, floating-gate memFETs may require relatively high gate pulse voltages, because charge must be injected through an insulating tunneling barrier.

On the other hand, ferroelectric materials have been incorporated into 2D FETs, relying on the residual polarization to modulate conductance with lower gate voltages on the order of 1 V. Chen et al. reported a WS_2_ FET with an Al_2_O_3_ capped HfZrO_2_ ferroelectric gate capable of emulating synaptic plasticity with a large memory window (Figure 4b) [95]. Similar works with other ferroelectric materials such as HfO_2_, CuInP_2_S_6_ (CIPS), α-In_2_Se_3_, PbZr_0.2_Ti_0.8_O_3_, and P(VDF-TrFE) have also recently reported synaptic capabilities [96,97,98,99,100,101]. Another approach for realizing artificial neurons specifically is to connect a 2D FET in series with a threshold-switching (TS) element, a volatile device that generates all-or-none spikes through abrupt resistive switching. Baek et al. demonstrated the use of a MoS_2_ FET connected to a ferroelectric CIPS TS device. Under a train of 2.0 V input pulses, Cu^+^ migration drove CIPS from its insulating ferroelectric state into a conductive ferroionic state, producing abrupt switching and LIF-like spike firing (Figure 4c). A device-performance based SNN simulation achieved 95.83% accuracy in face classification [102]. Although this approach effectively demonstrates spike firing, stochastic firing behavior and challenges in achieving reproducible responses may limit its appeal compared with other implementations. It should be noted that all of these reported 2D memFETs require complicated gate designs for the memory effect to be induced.

In contrast, electrolyte gating can provide low-voltage control by forming a molecular-scale electric double layer at the electrolyte/channel interface (Figure 4d). Electrolytes in the form of ionic liquids, ion gels, or solid polymer electrolytes are therefore attractive for use in artificial neurons and synapses. Shultz et al. demonstrated a 3 × 3 array of monolayer MoS_2_ memFETs gated by the ionic liquid DEME-TFSI. SET and RESET were induced using positive pulses of +1.0 to +2.0 V and negative pulses of −1.0 to −2.0 V, respectively [104]. The resulting memory states could be tuned by changing the pulse amplitude and duration. A device-performance based image-recognition demonstration achieved 96% accuracy using memFET-encoded data, in addition to spike firing when the memFET is connected to threshold-sensing circuit mimicking a leaky-integrate-and fire neuron. Table 1 compares the performance of selected memFETs in electrical mode. The reported neuromorphic tasks primarily involve pattern recognition, with neural-network performance evaluated through simulations using experimentally measured conductance states as synaptic weights.

#### 3.1.2. All Optically Operated 2d Memfets

In all-optically operated memFETs, both SET and RESET are induced by light, without electrical pulses. Bidirectional switching may arise from wavelength-dependent defects and trapping processes within a single semiconductor or from photoinduced charge transfer and polarization switching in a heterostructure. Ahmed et al. demonstrated a fully light-controlled memory phototransistor based on layered BP flakes with thicknesses of 5–25 nm on a SiO_2_/Si substrate. Native oxidation created P_x_O_γ_-related defects and trap states that produced a wavelength-dependent bidirectional photoresponse. Illumination at 280 nm generated a positive photocurrent and was used for optical SET and potentiation, whereas 365 nm illumination reduced the current and enabled optical RESET and depression. It should be noted that the photocurrents or photoresponses of 2D devices originate from the transistor channel conductance change and can be both positive and negative depending on the location of the so-called Dirac point, which is tunable by the applied gate voltage [111] and operation conditions [112]. An ANN simulation parameterized using the measured device characteristics achieved approximately 90% accuracy in MNIST handwritten-digit recognition [113].

**Figure 5:**
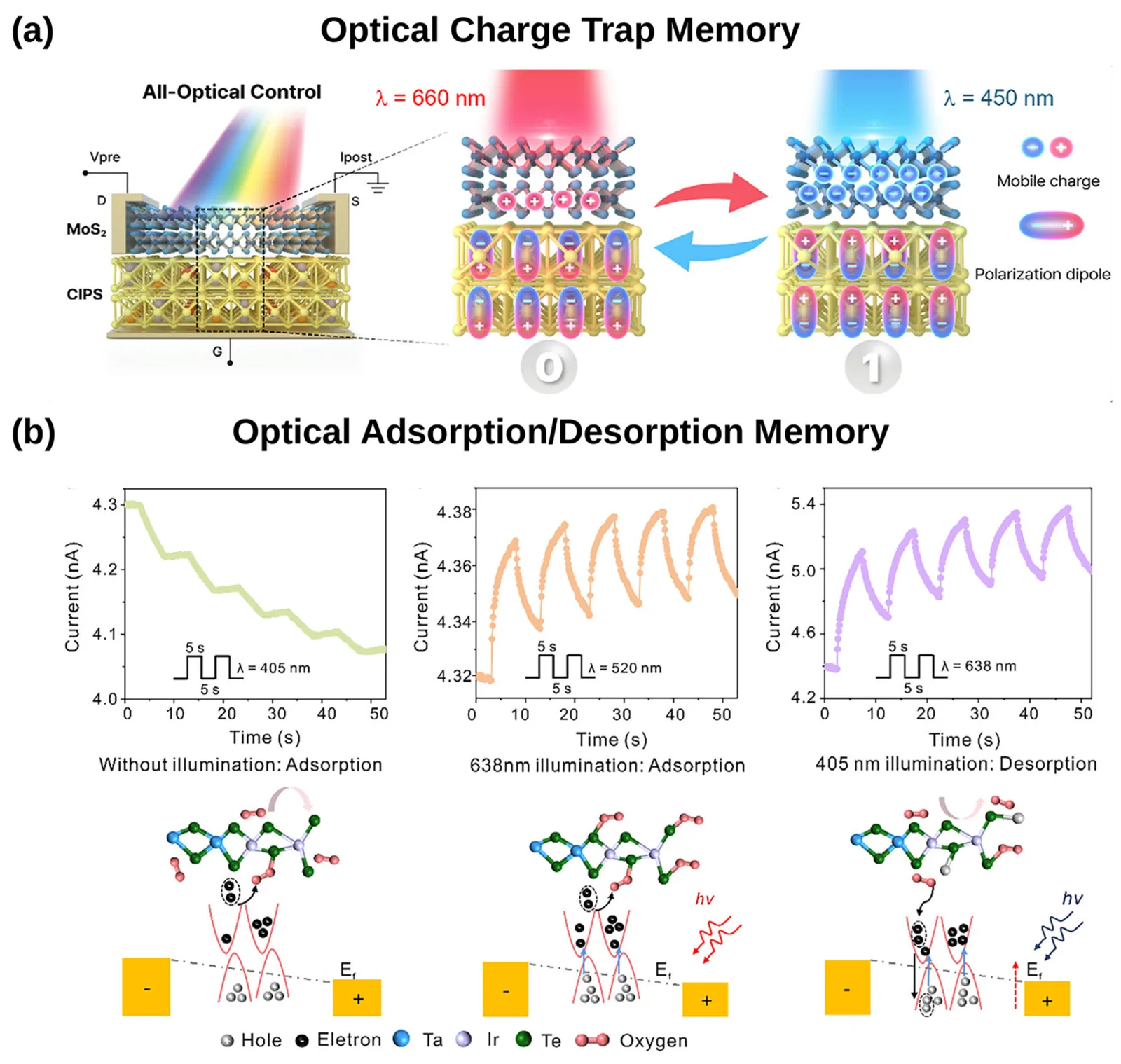
Optically Operated 2D memFETs: (**a**) Structure and mechanism of optical charge trap 2D memFETs. (**b**) Structure and mechanism of optical adsorption/desorption 2D memFETs. Copyright 2024, John Wiley and Sons. Adapted under CC BY 4.0 license [114]. Copyright 2024, John Wiley and Sons. Adapted with permission [116].

Niu et al. reported an all optically operated ferroelectric memory transistor based on a MoS_2_/CuInP_2_S_6_ (CIPS) van der Waals heterostructure (Figure 5a) [114]. Illumination at 660 nm wavelength induced potentiation and a high conductance state, whereas 450 nm wavelength illumination produced depression and a low-conductance state. Under 660 nm wavelength excitation, carriers were generated primarily in MoS_2_, and electron transfer into CIPS screened the interfacial polarization charge and stabilized upward polarization. Under 450 nm excitation, photocarriers were generated in both materials, and interfacial hole accumulation stabilized the opposite, downward polarization state. The device exhibited retention exceeding 10^4^ s and endurance exceeding 10^4^ switching cycles. An ANN simulation based on the measured potentiation and depression characteristics achieved 91.79% accuracy in MNIST recognition. Nevertheless, large-scale integration and array level uniformity remain important challenges for fully optical devices. Variations in material thickness, interface quality, and defect density can produce substantial device-to-device variability.

Another approach to 2D optical neuromorphic devices is optically stimulated adsorption/desorption of gas molecules to modulate channel conductivity. Liang et al. realized synaptic functionality in a TiO_2_ nanocrystal and graphene heterostructure, where O_2_ adsorption/desorption on TiO_2_ could be controlled via red/purple light [115]. The adsorbed O_2_ leads to a doping effect in the graphene, hence an alteration in the conductivity. A similar work using TaIrTe_4_ as the conductance channel reported adsorption/desorption with 638 nm/405 nm displaying similar synapse-like potentiation and depression with ultralow energy per pulse of 5.65 pJ (see Figure 5b) [116]. Other works take advantage of optical desorption and natural adsorption of ambient molecules for synaptic functionality. Stefano et al. reported an SnS_2_ based device achieving synaptic weights between 0.1 and 3000 [117].

#### 3.1.3. Optoelectronically Operated 2d Memfets

Optoelectronic 2D memFETs combine optical excitation with electrical control. In one common configuration, illumination SETs a memory state, whereas an electric gate pulse restores the initial current. In other devices, the gate continuously controls photocarrier trapping and detrapping, thereby tuning the duration of a light-induced state rather than producing a discrete RESET operation. Both device classes are relevant to neuromorphic vision, because conventional frame-based CMOS image sensors produce sequences of discrete frames and do not intrinsically encode temporal information. Optoelectronic devices with history dependent responses can instead combine sensing, temporal encoding, and preprocessing at the sensor level, supporting applications such as motion detection, trajectory analysis, and collision warning [118].

**Figure 6:**
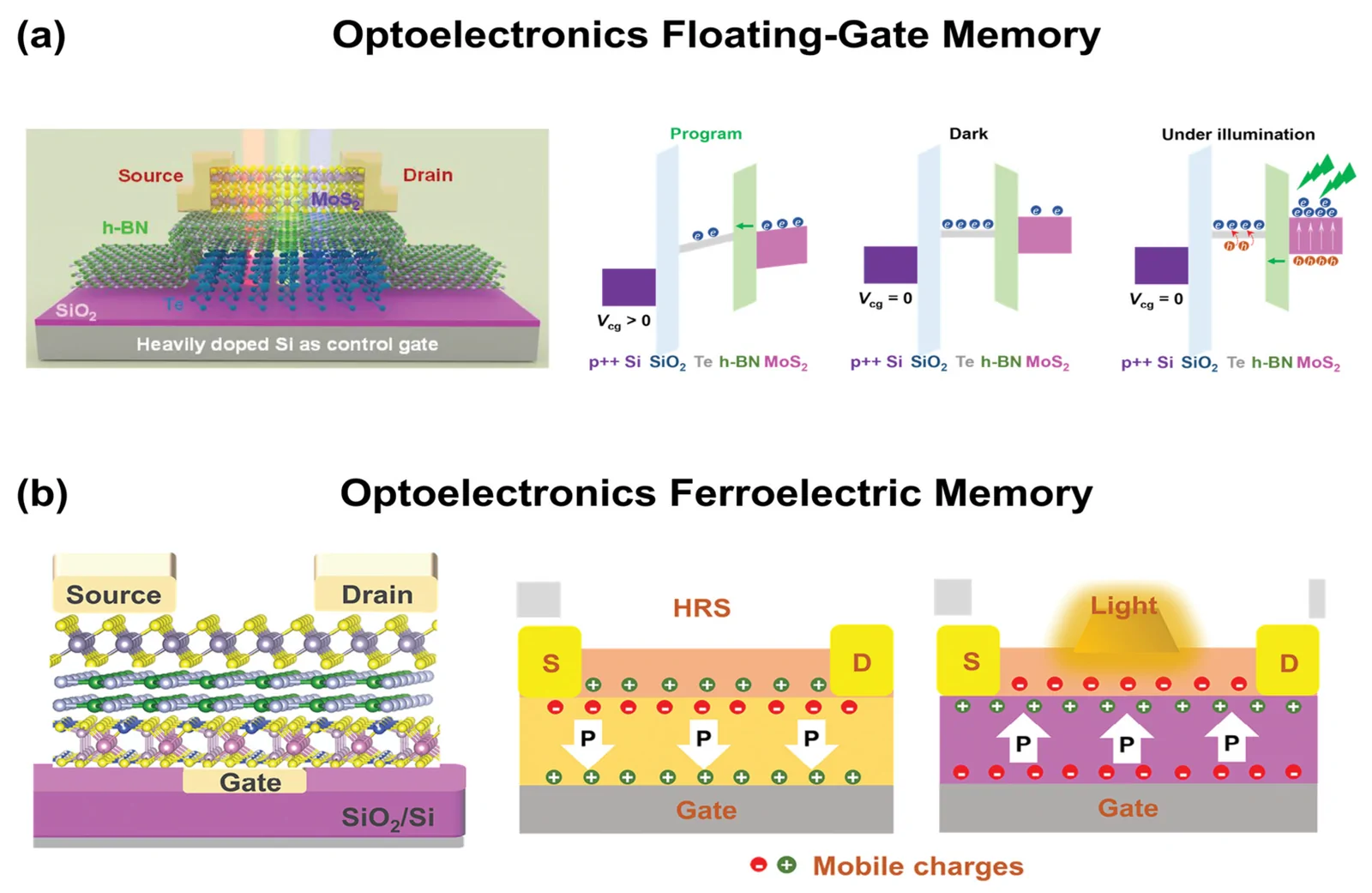
Optoelectronically Operated 2D memFETs: Device structures and mechanisms of combined electrical and optical memory operation in (**a**) MoS_2_/h-BN/Te floating-gate [121] and (**b**) SnS_2_/h-BN/CIPS ferroelectric memFETs [110]. Copyright 2023, John Wiley and Sons. Adapted under CC BY-NC 4.0 license [121]. Copyright 2023, John Wiley and Sons. Adapted under CC BY license [110].

Su et al. reported MoS_2_ memFET on a SiN_x_ trapping layer that combined image sensing with time-to-first-spike (TTFS) encoding. Photogenerated electrons were trapped and the charge-trapping layer held them too strongly to leak, thereby resulting in strong retention. A RESET pulse of +80 V, 1 s then restored the original conductance state. The devices distinguished accelerating, constant-speed, and decelerating motion. An SNN using the TTFS-encoded outputs achieved 98.86% recognition accuracy while reducing the number of spikes by 75% relative to rate coding [119]. However, strong retention in charge-trapping layers can suppress intrinsic leakage, producing a non-leaky neuronal response that requires a gate RESET pulse, potentially at high voltage, to restore the initial conductance. Chen et al. fabricated a 20 × 20 array of bilayer MoS_2_ memFETs with 40 nm Al_2_O_3_ local bottom-gate dielectrics. Under 660 nm illumination, trapping photogenerated carriers in intrinsic MoS_2_ gap states produced volatile temporal summation. Varying the gate voltage from +3 to −3 V tuned the decay time (retention), from approximately 10^1^ to 10^6^ ms. Positive gate bias inhibited electron trapping, whereas negative bias stabilized carriers in relatively deeper and longer-lived states. The array encoded left–right and approaching–receding motion, and a four-layer ANN achieved 99.2% accuracy in classifying five moving-ball actions [118]. Wang et al. demonstrated a homogeneously integrated neuromorphic-vision platform comprising a 1 × 4 array of few-layer MoS_2_ memFETs neurons and a 4 × 4 array of MoS_2_ ferroelectric (FeFET) synapses. The MoS_2_ memFETs neurons used a 30 nm Ti/Au local bottom gate and a 10 nm Al_2_O_3_ gate dielectric. 450 nm, 520 nm, and 650 nm optical SET pulses produced an accumulation of memory states, and when the accumulated conductance reached a predefined threshold, the memFET connected to the comparator circuit fired a spike. The FeFET synapses employed a 30 nm TiN/18 nm HZO/30 nm TiN/10 nm Al_2_O_3_/MoS_2_ gate stack and provided synaptic functions of multiple nonvolatile conductance states. An SNN simulation using the measured device characteristics achieved 91.7% accuracy in color recognition and 93.5% in object detection [120]. Electrical floating-gate and ferroelectric polarizations have also been combined with optical pulse in optoelectronics operations, as shown in Figure 6. Zha et al. used positive gate pulses to program a low-conductance state by storing electrons in the Te floating gate and light to neutralize the stored charge and increase the channel conductance (Figure 6a) [121]. Wang et al. used negative gate pulses to establish a low-conductance state by polarizing CIPS downward and light to reverse the polarization upward and switch the device into a high-conductance state (Figure 6b) [110]. These approaches enable optical SET and electrical RESET of the device conductance. However, both devices use electrical initialization before the optical SET. This additional conditioning step should be considered when evaluating the programming complexity and energy consumption of both approaches.

Table 2 summarizes the performance of several memFETs operating in all-optical or optoelectronic modes and briefly describes their memory mechanisms. Across many optoelectronic memFETs, a common operating scheme is optical SET followed by electrical RESET. The memory effect is typically realized through heterostructure engineering, in which different material components facilitate light absorption, carrier transfer, and charge trapping.

### 3.2. Implementation of 2d-Memfets in Neuromorphic Circuits

**Figure 7:**
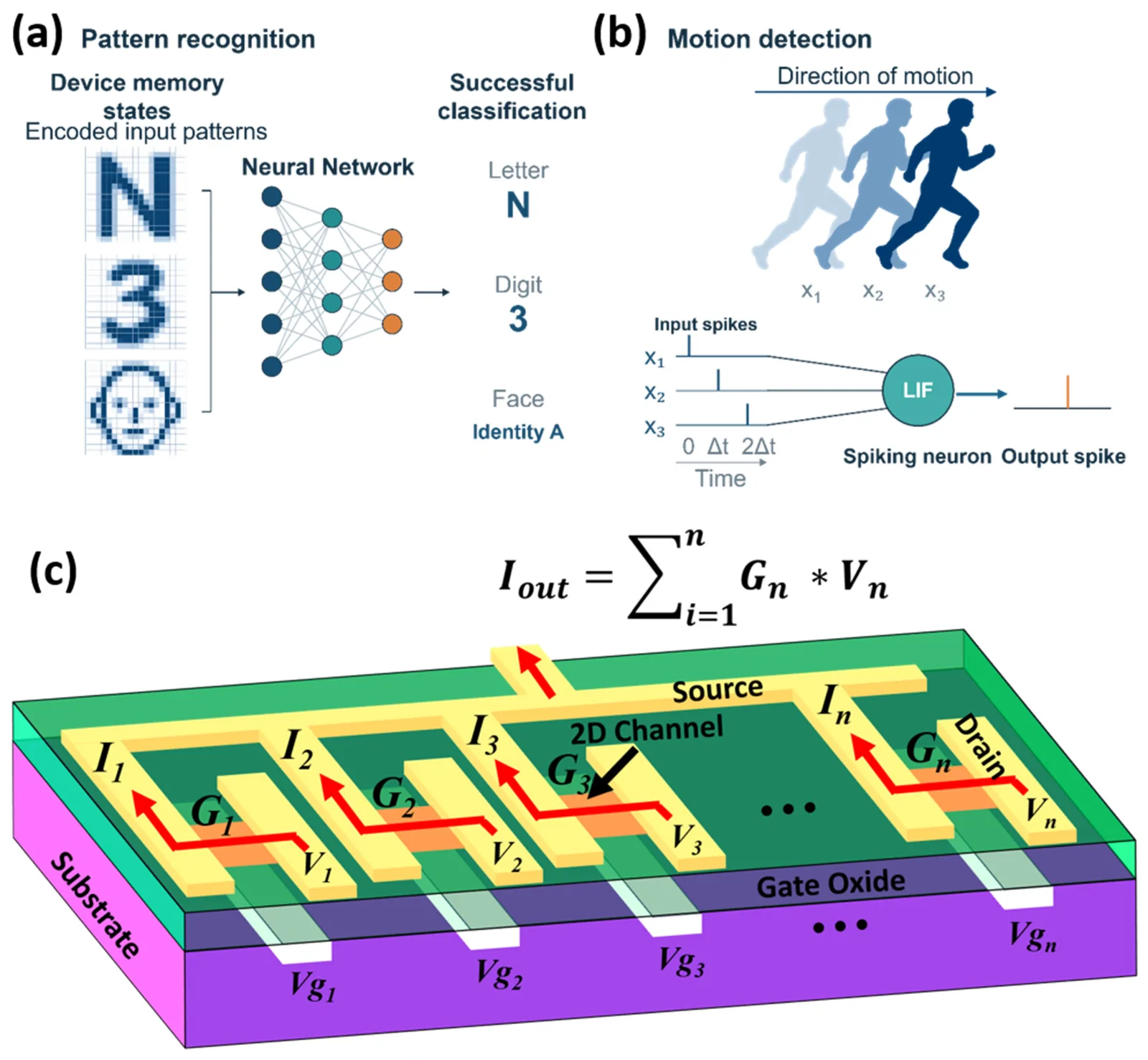
2D memFETs in Neuromorphic Circuits: (**a**) Pattern recognition using distinct device memory states to encode letters, digits, or faces for classification by trained neural networks such as ANNs and CNNs. (**b**) Motion detection based on the relative timing of input spikes from different spatial locations. A neuron integrates these inputs and generates an output spike when its firing threshold is reached. (**c**) Diagram of multiple memFETs arranged as synaptic weights outputting a weighted average current based on their combined input signals (voltages) and channel conductance.

As was previously stated, 2D memFETs must perform consistently and have a high uniformity in arrays of devices in order to be considered for neuromorphic computing. Often, researchers will demonstrate a single artificial neuron or synapses based on 2D FETs, then extrapolate these results into a full simulation including hundreds to thousands of these devices working together. Although this is a useful intermediate step, the reality of creating even a small array of uniform 2D memFETs is nontrivial, and reported simulated accuracies of >97% are likely not taking into account real-world nonuniformity. In Figure 7a, distinct device memory states encode static information for classification by a trained neural network, enabling recognition of letters, digits, and faces using publicly available datasets such as the Yale Face Database and MNIST. In Figure 7b, an SNN model processes motion information encoded in the timing of sequential input spikes from different spatial positions. LIF neurons integrate these inputs over time and fire when their thresholds are reached. The SNN can classify motion and direction, for example by extracting temporal correlations between events, although this can be extrapolated to any time-based classification problems such as voice recognition or signal analysis [125]. For the weighted summation of neural network signals for neuron processing or classification, memFETs functioning as synapses are connected in parallel with a common electrode to act as a summing point for currents (Figure 7c). These channel conductances attenuate pulse or spike signals, which output as the summed and weighted train of signals that can be sent to an LIF neuron.

Marega et al. [126] demonstrated an array of seven floating gate monolayer MoS_2_ FETs with memory characteristics. As the control gate voltage was applied, the bands in the dielectric gate stack were bent, allowing direct tunneling through HfO_2_ from the MoS_2_ channel into a Pt floating gate, thus changing the threshold voltage and altering the channel conductance, which may be programmed with up to 16 states (4-bit). As a test of ANN classification, the seven pips that make up a digital numeral were used as the dataset to classify the numbers 1, 4, and 6 using a softmax output function. Interestingly, the group was able to program all seven memFETs into a similar range of channel conductance using a dynamic pulsing amplitude and write and verify scheme to ensure the correct weight was set for each device. Hardware level VMM was carried out by connecting all of the memFETs together at their source electrodes and inputting pattern data into the drain electrodes of all seven channels simultaneously, then reading the summed output current, which was then processed numerically ex situ (Figure 7c). On hardware, a max recognition rate of 91.5% was recorded compared to the simulated 95.5%, with some tests resulting in accuracy as low as 80%. However, to accomplish this, the same seven memFETs had to be reused and reprogrammed for each specific digit. Furthermore, weights were calculated ex situ and imported, with no in situ training to account for device nonidealities, which could increase accuracy but would also use significant amounts of energy. Although producing seven memFETs with similar channel properties is an impressive display of 2D device uniformity, normal VMM is carried out simultaneously, which in this case would require three sets of seven uniform memFETs. This not only would increase uniformity restrictions but would also drastically increase fabrication complexity with a need to isolate multiple layers of wires from one another.

Won et al. [127] created a tunable floating gate memFET using monolayer MoS_2_ as the FET channel, graphene as the floating gate, and Al_2_O_3_ as the gate dielectric, with the capability to act as both a synapse or an LIF neuron. At thicker gate dielectric thicknesses of ~8 nm, charge cannot leak to/from the floating gate, allowing non-volatile retention of each memFET’s channel conductance. On the other hand, a thinner ~ 4 nm gate dielectric allows tunneling from the floating gate, enabling volatile LIF characteristics. These LIF neurons were combined with a comparator outputting a negative bias when a threshold was surpassed, causing automated resetting of the neuron’s floating gate, providing an output spike that could be used for classification or future network layers. A third use for this output feeding back into the non-volatile synapses is to enable brain-like spiking time dependent plasticity (STDP) by lowering the potential difference seen by the memFET. A dataset of three images that were 3 × 3 pixels were used to carry out supervised learning using STDP, resulting in accurate recognition of the three classes of images. This work is of particular significance due to the full functionality and mostly monolithic (besides the added comparators) integration of artificial synapses and neurons allowing consistent synaptic programming of 27 2D memFETs and three neurons firing with similar inputs, while also providing its own automated resetting mechanism, which is critical for accurate computation.

## 4. Summary Remarks and Future Perspectives

In summary, the need for high-density, energy-efficient memory devices for in-memory computing has driven the research and development of 2D memFETs, which will continue its upward trajectory. This trend can be regarded as a part of the empirical Moore’s law toward smaller devices in microelectronics for advanced computing during the past five decades. The future needs of AI-based microelectronics and computing will drive the research into 2D memFETs, seeking their implementation into practical circuits. Nevertheless, many challenges to the integration of 2D memFETs must be addressed before large-scale NC circuits applications may be realized. A question arises about fundamental obstacles that must be overcome to enable practical circuit applications. Four of them are listed below based on their priorities. First and most importantly, different control mechanisms, such as electrical, optical, optoelectronic, mechanical, etc., must be explored for controllable generation and switching memory states. Considering 2D memFETs are dynamic devices, it is particularly important to control the switching speed, retention, endurance, and switching energy efficiency. Furthermore, for application of the 2D memFETs as artificial neurons in SNN-based neuromorphic computing, the demonstration of LIF characteristics is imperative, which requires further control of the switching dynamics of the 2D memFETs. Moreover, it has been demonstrated that peripheral CMOS circuitry involved in driving or converting analog signals from artificial synapses and neurons (ADC/DAC) makes up over 90% of the overall power usage of memristive NC systems, demonstrating a need for fewer and more efficient digital/analog conversions to deal with the massive energy overhead and take full advantage of energy-efficient 2D memFETs [30]. In fact, a matrix for the evaluation of key device properties of 2D memory transistors in comparison to CMOS-based ones, including switching speed, switching energy, retention, endurance, yield, and uniformity, needs to be established. Such a metric is critical to NC circuit design and scalability. Finally, large arrays of 2D memFETs with suitable interfaces with existing Si-based readout circuits must be achieved. Multiple issues in the synthesis of monolayer 2D atomic materials and VDWH, including requirements of lateral dimension and low tolerances for the defects that may form during the synthesis, transfer, and fabrication of devices. It should be noted that this is by no means trivial. While CVD growth of certain 2D materials such as graphene and TMDCs has been reported successful in large wafers, exfoliation and transfer of the CVD 2D materials from substrates used for CVD growth to those used for devices are cumbersome at circuit scales. Therefore, intensive research on the development of 2D memFETs will remain a focus of many groups focused on resolving these challenges and critical issues in future research.

## Figures and Tables

**Table 1:** Performance of electrically operated memFETs. Abbreviations used include: ANN: Artificial Neural Network, MNIST: Modified National Institute of Standards and Technology Database, and CNN: Convolutional Neural Network. NA regards “not apply” since data is not available.

Device Structure	Memory Mechanism	SET/RESET	Retention	Endurance	Memory Switching Ratio	Memory Switching Energy (SET/RESET)	Neuromorphic Task
Monolayer MoS_2_/oxide tunnel barrier/multilayer-graphene floating gate (Ref. [105])	Fowler–Nordheim charge tunnelling to and from graphene	−18 V, 100 ms/+18 V, 100 ms	>10^4^ s	>120 cycles	>10^4^	NA	NA
hBN/α-In_2_Se_3_/hBN ferroelectric-channel transistor (Ref. [106])	Reversal of ferroelectric polarization in the semiconductor channel	−8 V, 10 s/+8 V, 10 s	≈500 s	500 cycles	>10^3^	234 fJ/40 fJ	ANN iris classification, 94.74%
MoS_2_/hBN/MoS_2_ floating gate/GDYO threshold-switching layer/WSe_2_ (Ref. [107])	Direct charge injection through a volatile threshold-switching layer	+2 V, 20 ns/−2 V, 20 ns	>10^4^ s	>10^3^ cycles	10^7^	10 fJ/10 fJ	NA
Monolayer WS_2_/Na^+^-diffused SiO_2_ solid-state ionotronic FET (Ref. [108])	Field-driven Na^+^ migration and interfacial electrostatic doping	+50 V, 4 s/−50 V, 4 s	>10^3^ s	>100 sweep cycles	>10^6^ (at elevated temperature)	<1 pJ (at elevated temperature)	ANN MNIST recognition > 90% after 20 epochs
R-stacked bilayer MoS_2_ sliding-ferroelectric FET on flexible PET (Ref. [109])	Sliding ferroelectric polarization switching	−10 V, 100 ms/+10 V, 100 ms	≈10^4^ s	>10^4^ switching cycles	>10^6^	33.9 pJ/28.7 pJ	CNN MNIST accuracy 97.81%
2D α-In_2_Se_3_ channel/SiO_2_ dielectric (Ref. [99])	Band bending caused by polarization-bound charges	−5 V, 0.05 s/+5 V, 0.5 s	600 s for SET pulse −60 V/0.5 s	NA	NA	40 pJ/40 pJ	ANN MNIST accuracy 94%
SnS_2_/h-BN/CuInP_2_S_6_ (Ref. [110])	Downward CuInP_2_S_6_ polarization depletes electrons in the SnS_2_ channel, while upward polarization accumulates electrons in the channel.	+25 V, 10 s/−25 V, 10 s	>10^4^ s	350	10^5^	NA	NA

**Table 2:** Performance of optically and optoelectronically operated 2D memFETs. NA regards “not apply” since data is not available.

Device Structure	Operating Mode	Memory Mechanism	SET/RESET	Retention/Endurance	Memory Switching Ratio	Memory Switching Energy (SET/RESET)	Neuromorphic Task
Monolayer WSe_2_/hBN memFET (Ref. [122])	Optoelectronics	Photogenerated electrons transfer from BN’s mid-gap states into WSe_2_ leaving behind trapped positive charge in the BN layer.	405 nm illumination with −80 V gate bias	NA for optical	>10^6^	1.0 nJ (optical SET)	NA
Oxidized layered-black phosphorus memFET (Ref. [113])	All optical	Wavelength-selective positive and negative photoconductivity	280 nm/365 nm	NA	NA	NA	image recognition > 90%
MoS_2_/hBN/Te floating-gate FET (Ref. [121])	Optoelectronics	Charge storage in Te	−12 V, 50 ns or 532 nm/+12 V, 500 ns	>10^3^ s optical retention	>10^5^	NA	Reservoir computing: N-MNIST recognition 90.77%
SnS_2_/hBN/CuInP_2_S_6_ photoferroelectric FET (Ref. [110])	Optoelectronics	SnS_2_ photocarriers trigger CuInP_2_S_6_ polarization reversal	457 nm/−5 V, 0.1 s	10^2^ s	NA	NA	Reservoir computing: MNIST accuracy 93.62%
MoS_2_/Al_2_O_3/_Au NP as Floating Gate/Al_2_O_3_ (Ref. [123])	Optoelectronics	Charge storage in the Au nanoparticles Floating-Gate	457 nm, 532 nm, 650 nm/up to 15 V	2000 s	>10^4^	416 pJ	Object recognition using R-CNN
Te/SnS_2_ (Ref. [124])	Optoelectronics	Heterojunction charge separation and vacancy trapping	532 nm while −60 V gate voltage applied simultaneously/60 V pulse to RESET the device	1200 s	NA	NA	ANN: MNIST recognition accuracy up to 94.4%

## Data Availability

No new data were created or analyzed in this study. Data sharing is not applicable to this article.
